# LIF and Oncostatin M mitigate LPS-induced acute endometritis via distinct immunoregulatory mechanisms in peritoneal macrophages *in vivo*

**DOI:** 10.1016/j.isci.2026.116046

**Published:** 2026-05-27

**Authors:** Marion Ravelojaona, Julie Girouard, Cathy Vaillancourt, Céline Van Themsche, Carlos Reyes-Moreno

**Affiliations:** 1 Département de Biologie Médicale, Université du Québec à Trois-Rivières, 3351 Boul. des Forges, Trois-Rivières, QC G8Z 4M3, Canada; 2 Centre de Recherche Interuniversitaire en Reproduction et Développement-Réseau Québécois en Reproduction (CIRD-RQR), Université de Montréal, St-Hyacinthe, QC J2S 2M2, Canada; 3 Regroupement Intersectoriel de Recherche en Santé de l’Université du Québec (RISUQ), Université du Québec, Québec, QC G1K 9H7, Canada; 4 Centre Armand Frappier Santé Biotechnologie, Institut National de la Recherche Scientifique (INRS), Laval, QC H7V 1B7, Canada

**Keywords:** Microenvironment, Immunology, Cancer

## Abstract

Acute endometritis, often triggered by Gram-negative bacterial endotoxins such as lipopolysaccharide (LPS), disrupts endometrial integrity, induces robust pro-inflammatory macrophage activation, and compromises uterine function, potentially leading to infertility and adverse pregnancy outcomes. Here, we demonstrate that IL-6 family cytokines, Leukemia Inhibitory Factor (LIF) and Oncostatin M (OSM), exert complementary yet distinct immunoregulatory effects that mitigate LPS-induced acute endometritis *in vivo*. Both cytokines preserved endometrial architecture, limited neutrophil and macrophage infiltration, suppressed cytotoxic iNOS expression, and promoted macrophage-mediated tissue repair, with OSM providing superior systemic and hepatoprotective effects. Mechanistically, these outcomes were linked to STAT3-dependent Mφ reprogramming toward anti-inflammatory M2 phenotypes, with OSM additionally engaging SMAD2 pathways to favor CD163 M2 macrophages marker, whereas LIF preferentially induced anti-inflammatory M2 subsets engaging STAT3 alone. Our findings highlight the therapeutic potential of LIF and OSM as modulators of uterine immune homeostasis and macrophage plasticity modulating and mitigating LPS-acute endometritis and preserving endometrial integrity.

## Highlights

• LIF and OSM maintain endometrium and uterine gland integrity during inflammation
• LIF and OSM reprogram macrophages via distinct anti-inflammatory pathways
• OSM activates SMAD2 signaling, inducing CD163 and protecting liver tissue
• LIF and OSM may enhance IVF outcomes and reduce endometritis, with or without antibiotics

## Graphical abstract

**Figure:**
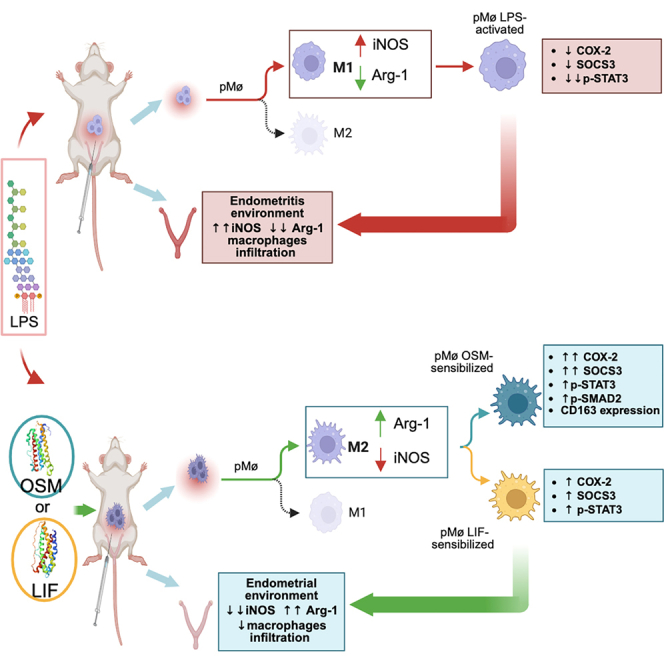

## Introduction

Endotoxin, such as lipopolysaccharide (LPS), is a major component of Gram-negative bacteria. Its presence in normally sterile compartments such as the uterine cavity indicates microbial invasion, and elevated levels in cervical mucus or vaginal secretions reflect an increased Gram-negative microbial burden commonly associated with bacterial vaginosis.^1^ Bacterial vaginosis, affecting approximately 11–15% of non-pregnant women, is regarded as a significant risk factor for first-trimester miscarriage, spontaneous preterm birth, and the development of postpartum endometritis.^1^ In most cases, intrauterine endotoxin results from ascending Gram-negative bacterial colonization, although inadvertent introduction during *in vitro* fertilization (IVF) procedures has also been suggested.^1^ Studies report that women undergoing IVF with elevated intrauterine endotoxin levels (>200 pg/mL) fail to achieve pregnancy, underscoring the detrimental impact of endotoxin on implantation and pregnancy outcome.^1^ Within the uterine cavity, endotoxins such as LPS rapidly induce a robust inflammatory response characterized by neutrophil infiltration, microabscess formation, and disruption of the endometrial microenvironment, all hallmarks of acute endometritis.^2^^,^^3^ In preclinical models of acute endometritis, LPS administration generates extensive inflammatory cell infiltration to uterine tissue and enhanced expression of several pro-inflammatory factors.^4^^,^^5^

Notably, endometritis, if not promptly diagnosed and treated, can lead to long-term complications and infertility^6^ by reducing endometrial receptivity and impairing embryo implantation.^2^^,^^7^ Bacterial products, along with macrophages (Mϕs) and Th1 mediators, contribute to the mechanisms underlying pregnancy disorders linked to endometrial inflammation.^1^ In fact, Mϕs are essential for endometrial integrity, uterine receptivity, and innate immune protection.^8^ In response to inflammatory stimuli, Mφs engage a network of intracellular signaling pathways that determine their polarization toward M1 or M2 states, depending on the context of activation.^9^ Upon sensing bacterial infection or endotoxin, TLR-mediated activation drives a rapid shift toward a pro-inflammatory M1 phenotype, marked by the release of interleukin (IL)-1β, IL-6, tumor necrosis factor α (TNFα), interferon (IFN)-γ, and granulocyte-macrophage colony-stimulating factor (GM-CSF).^8^^,^^10^ In a mouse model, this response can be amplified by nitric oxide (NO) and reactive oxygen species (ROS) production, which contribute to tissue injury.^11^ Prolonged NO production, however, can cause tissue damage including vascular dilation, sepsis, and disruption of the uterine environment.^12^ Because the endometrium normally maintains a tolerogenic, Th2-dominant environment, timely reprogramming of Mϕ toward an M2 phenotype is critical for tissue repair and inflammatory resolution.^8^ Immunomodulatory factors have been identified as key drivers of intrauterine inflammation resolution, preventing pregnancy complications such as preterm birth and promoting M2 polarization in gestational tissues.^13^ Failure to restore this balance can lead to persistent inflammation, endometrial dysfunction, and fertility impairment.^14^^,^^15^

Within this framework, STAT3 has emerged as a pivotal immunoregulatory transcription factor. Activated downstream of IL-6 family cytokines, including Leukemia Inhibitory Factor (LIF) and Oncostatin M (OSM), STAT3 signaling promotes the resolution of inflammation by driving Mφ polarization toward the M2 phenotype, also referred to as the alternative activation state.^16^^,^^17^ LIF mitigates LPS-induced inflammation and complements the anti-inflammatory actions of progesterone in the uterine environment.^18^ Mechanistically, our previous study demonstrated that LIF regulates Mϕ responses through STAT3 activation and IL-10 production by syncytiotrophoblasts, thereby suppressing the pro-inflammatory STAT1 and STAT5 pathways triggered by IFN-γ and GM-CSF.^16^^,^^19^ In parallel, in trophoblasts, our previous work further demonstrated that OSM-dependent STAT3 signaling regulates β-hCG production and modulates IFNγ-STAT1 and GM-CSF-STAT5 pathways, with potential STAT3-independent contributions involving TGFβ1-SMAD2 and SOCS1/3.^17^

Building on our previous *in vitro* work identifying how these cytokines modulate Mϕ signaling pathways to restrain excessive inflammatory activation, the present study aimed to delineate the mechanisms through which LIF and OSM regulate the pro-inflammatory activity of peritoneal Mϕs in response to LPS *in vivo*. While the anti-inflammatory effects of LIF during LPS-induced intrauterine inflammation are relatively well established, the intrauterine functions of OSM in this context remain less explored. Therefore, this study investigates not only the immunoregulatory and tissue-protective effects of LIF and OSM in a mouse model of LPS-induced endometritis, but also the molecular pathways through which these cytokines orchestrate Mϕ-mediated inflammatory control and promote endometrial repair.

## Results

### LIF and OSM have a protective effect on body weight loss induced by LPS-related inflammatory response

**Figure 1:**
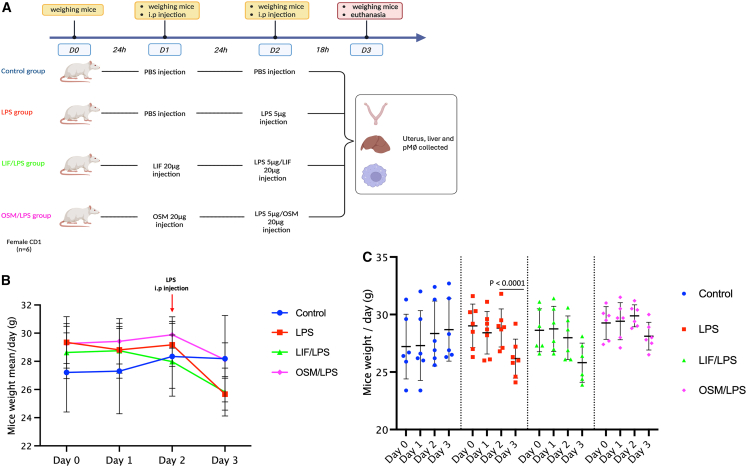
LIF and OSM prevent the loss of body weight in mice induced by LPS administration (A) Experimental design of mice experimental protocol. Mice at day 0 (D0) were weighed and separated into four groups. Group 1: Control; Group 2: LPS; Group 3: LIF/LPS; and Group 4: OSM/LPS. The Control group were injected with PBS as control vehicle. Recombinant LIF or OSM proteins were administrated at 20 μg/mouse, representing 560 μg/kg per mouse, to the LIF/LPS or OSM/LPS groups, respectively, by intraperitoneal (i.p.) injections; at D2, the LPS group received the LPS i.p. injection at 5 μg/mouse, representing 140 μg/kg per mouse. Co-injections of LPS with LIF or OSM were administrated to the LIF/LPS or OSM/LPS groups by i.p. injections. Mice groups at D3 were weighed, euthanized, then peritoneal Mφs (p Mφs), liver, and uterus were collected. (*n* = 6 per group). (B) Curve of mouse mean weight/day in different groups. (C) Graph of mouse mean weight in different groups at day 0, day 1, day 2, and day 3 before euthanasia. Data are presented as mean ± SEM. Error bars represent SEM. Statistical analysis was performed using Student’s *t* test and Wilcoxon test.

Intraperitoneal (i.p.) administration of LPS at 140 μg/kg body weight (BW) in mice induces acute inflammation and reduces BW.^20^ This dose corresponds to approximately 5 μg of LPS per mouse, based on an average weight of 28 g. To assess the potential of LIF or OSM in protecting BW during LPS-induced inflammation, we conducted a randomized study with mice distributed into four groups. Mice were weighed daily before and after intraperitoneal injections of LIF or OSM, in addition to LPS administration (Figure 1A).

At D0, the mean BW was similar across groups: control 27.2 ± 2.8 g, LPS 29.0 ± 1.7 g, LIF/LPS 28.6 ± 1.9 g, and OSM/LPS 29.3 ± 1.4 g (Figure 1B). On day 1 (D1), mice received 0.1 mL i.p. injections of LIF or OSM at 20 μg/mouse for the respective groups, while control and LPS groups received 0.1 mL PBS. No significant BW changes were observed compared with D0 (Figures 1A and 1B). On day 2 (D2), BW changes were minor and not statistically significant: control +4.1%, LPS +1.5%, LIF/LPS -3%, OSM/LPS +1.6% (Figure 1B). After injections on D2, control mice received PBS, the LPS group received 5 μg LPS/mouse, and the LIF/LPS and OSM/LPS groups received LIF or OSM co-administered with LPS (Figure 1A). On day 3 (D3), prior to euthanasia, BW was measured again. The LPS group showed a significant reduction (−9%), from 28.8 ± 1.1 g on D2 to 26.2 ± 1.1 g on D3. In contrast, BW reductions in the LIF/LPS (−8%) and OSM/LPS (−6%) groups were not statistically significant (Figure 1B). These results suggest that LIF and OSM may partially protect against LPS-induced weight loss.

This analysis confirms that i.p. injection of LPS induces acute systemic inflammation in our murine model. Consistent with previous studies, systemic LPS triggers immune activation and metabolic alterations, including rapid BW reduction due to TLR4-mediated lipolysis in visceral adipose tissue.^21^^,^^22^^,^^23^ Our data show a pronounced decrease in BW 24 h after LPS administration in the LPS-only group, whereas co-administration of LIF or OSM attenuated this weight loss (Figure 1C). This indicates that both cytokines can mitigate LPS-driven metabolic effects *in vivo*.

Although the molecular mechanisms were not directly investigated here, IL-6 family cytokines such as LIF and OSM are known to regulate adipocyte function and differentiation via LIFR and OSMR, signaling through the gp130 co-receptor and activating STAT3 pathways.^24^^,^^25^^,^^26^ Prior studies have suggested links between these cytokines and BW regulation, but their role in immunometabolic responses during acute inflammation remains underexplored.

### LIF and OSM exert distinct modulatory effects on LPS-induced acute inflammation in uterine and liver tissues

**Figure 2:**
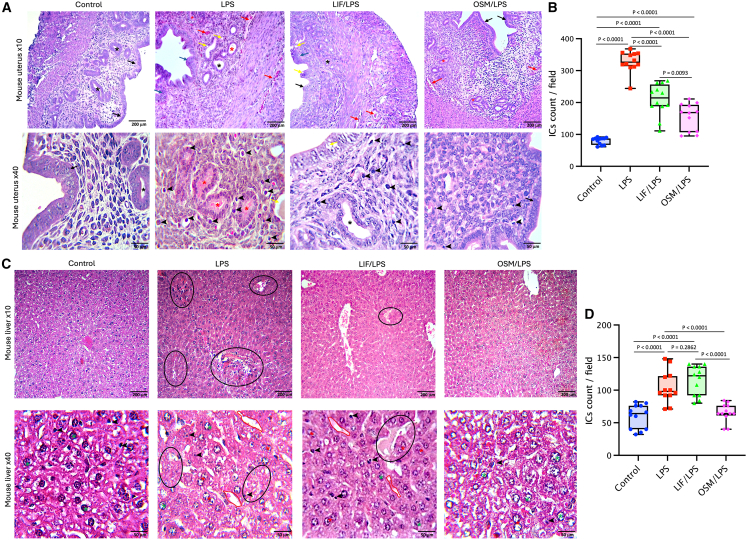
LIF or OSM injections mitigate LPS-induced inflammation and inflammatory cell (IC) infiltration in uterine and liver tissues (A) H&E-assisted histopathological analysis of uterine tissues from Control, LPS, LIF/LPS, and OSM/LPS groups (original magnification ×10 and x40). Black arrow: simple columnar epithelium; black asterisk: uterine glands; blue arrow: hypercellular layer of columnar epithelium; red arrow: hemorrhagic areas; black arrowhead: IC-infiltrated areas; red asterisk: cystic endometrial glands; yellow arrow: apoptotic necrosis areas; black arrow: healthier endometrial tissue. (B) Graphical analysis of IC counts per field in uterine tissues from the different groups. (C) H&E-assisted histopathological analysis of liver tissues from Control, LPS, LIF/LPS, and OSM/LPS mice groups (original magnification ×10 [scale bars: 200 μm] and x40 (scale bars: 50 μm]). Green asterisk: mononuclear hepatocytes cells; black arrowhead: ICs; black circle: necrotic zone or area of hepatocyte degeneration; red asterisk: binuclear hepatocytes; red line: expansion of sinusoids; black circle: spotty necrosis. (D) Graphical analysis of IC counts per field in liver tissues from the different groups. Data are presented as mean ± SEM. Error bars represent SEM (*n* = 12 fields per group). Statistical analysis was performed using unpaired Student’s *t* test.

Given the close interplay between lipid metabolism, inflammation, and hepatic function,^21^ we also analyzed liver tissue in addition to uterine tissue. Acute LPS exposure induces hepatocyte necrosis and apoptosis, contributing to liver injury and sepsis. LPS-triggered systemic inflammation can affect multiple organs, including the liver and uterus. During such inflammation, hepatic synthesis of IL-6 family cytokines, including LIF and OSM, plays a crucial role in maintaining homeostasis, promoting repair, and preserving tissue integrity.^27^^,^^28^ To investigate both local (uterine) and systemic (hepatic) effects of LIF and OSM, we performed a dual-tissue analysis. The uterus and liver from mice in each group were collected, and histopathological changes were examined after H&E staining at 10× and 40× magnifications (Figures 2A and 2C). Semi-quantitative histopathological scoring of uterine and liver tissues are decribed in Table 1.

**Table 1:** Semi-quantitative histopathological scoring of uterine and liver tissues

	Control	LPS	LIF/LPS	OSM/LPS
Epithelial integrity	0	3	2	1
Glandular structure	0	3	2	2
Hemorrhage	0	3	2	1
Inflammatory cell (IC) infiltration	0	3	3	1
Apoptosis/Necrosis	0	3	2	1
Mononuclear hepatocytes	3	2	3	2
IC infiltration	0	3	3	1
Sinusoid expansion	0	3	3	1
Necrotic areas	0	3	2	1
Binuclear hepatocytes	0	3	2	2

**Scoring scale:** 0 = absent/normal tissue; 1 = mild alteration; 2 = moderate alteration; 3 = severe alteration.

#### Uterine histopathology

At low magnification (10×), control uteri displayed intact endometrium with well-defined epithelial and stromal layers. Endometrial glands were well developed, and glandular epithelial cells showed regular columnar or cuboidal morphology (Figure 2A, 10×; black asterisk and arrow). In LPS-treated uteri, tissue damage was evident. Necrotic areas and edema disrupted tissue architecture (yellow arrow), and glandular structures appeared irregular (red asterisk). Inflammatory and red blood cells infiltrated the endometrial and myometrial layers (red arrows; Figure 2A, 10×). At higher magnification (40×), vascular congestion, epithelial alterations, necrotic foci, pus-filled glands, and dense inflammatory infiltrates were apparent (red, blue, yellow arrows; black arrowhead; Figure 2A, 40×). Remarkably, LIF and OSM attenuated these histopathological changes. In particular, OSM/LPS-treated mice retained a more regular epithelial layer and glandular epithelium, with fewer necrotic and hemorrhagic areas and reduced inflammatory cell infiltration (black arrow; black arrowhead; Figure 2A, 40×). Quantification of inflammatory cells (ICs) confirmed these observations (Figure 2B). Control uteri had sparse ICs (80 ± 11 cells/field). LPS induced a ∼4-fold increase (328 ± 33 cells/field). LIF/LPS and OSM/LPS groups showed statistically significant reductions, averaging 209 ± 50 and 155 ± 43 cells/field, respectively (∼2-fold decrease). These results indicate that both cytokines protect the uterine microenvironment during acute endotoxemia, with OSM showing slightly stronger preservation of tissue integrity.

#### Hepatic histopathology

Intraperitoneal LPS injection has been shown to simulate exogenous infection and induce hepatic inflammation.^28^ Conversely, IL-6 family cytokines such as LIF and OSM are produced in the liver during acute inflammation and participate in tissue-protective responses by promoting regenerative pathways and maintaining tissue homeostasis.^29^

Control livers exhibited normal lobular architecture, regular sinusoidal spaces, and uniform hepatocytes with minimal ICs (green asterisk; black arrowhead; Figure 2C). LPS induced necrosis, hepatocyte ballooning, binucleation, and sinusoidal expansion with hemorrhagic lesions (black circle; red line; Figure 2C). IC infiltration increased ∼2-fold (104 ± 25 cells/field vs. 60 ± 18 in controls; Figure 2D). The LIF/LPS group showed similar IC infiltration (115 ± 23 cells/field). In contrast, OSM/LPS mice had markedly reduced IC numbers (65 ± 18 cells/field), comparable with controls, indicating better systemic protection. Interestingly, OSM effectively mitigates acute hepatic inflammation, whereas LIF shows limited hepatic protection under acute LPS challenge.

Overall, this immunohistochemical analysis shows that LPS induces both local uterine inflammation and systemic hepatic injury. LIF and OSM both reduce histopathological damage and IC infiltration in uterine tissues, whereas OSM but not LIF additionally protects the liver, highlighting tissue-specific differences in cytokine efficacy. These findings support a broader immunoregulatory role for IL-6 family cytokines during acute inflammation.

### LIF and OSM regulate iNOS, Arg-1 expression, and Mϕ infiltration in the uterine environment during LPS-induced acute inflammation

LPS administration elicits a strong innate immune response characterized by the production of pro-inflammatory mediators, including TNF-α, IL-1, IL-6, and NO, predominantly by activated Mϕs.^30^ NO plays a pivotal role in antimicrobial defense mechanisms, including the elimination of bacteria, viruses, and tumor cells. However, its interaction with ROS leads to the formation of peroxynitrite, a potent oxidant contributing to tissue inflammation and injury.^30^ The enzyme inducible nitric oxide synthase (iNOS) catalyzes NO synthesis from L-arginine, whereas Arg-1, another enzyme involved in L-arginine metabolism, converts this amino acid into L-ornithine and urea, thereby limiting substrate availability for NO production.^31^^,^^32^ To assess the inflammatory status of uterine tissue following acute LPS exposure, we therefore evaluated the expression of iNOS and Arg-1 proteins, alongside the detection of Mϕ populations. These analyses enabled us to determine the extent of inflammation and Mϕ activation in response to LPS, and to compare these effects with those observed upon sensitization with LIF or OSM.

**Figure 3:**
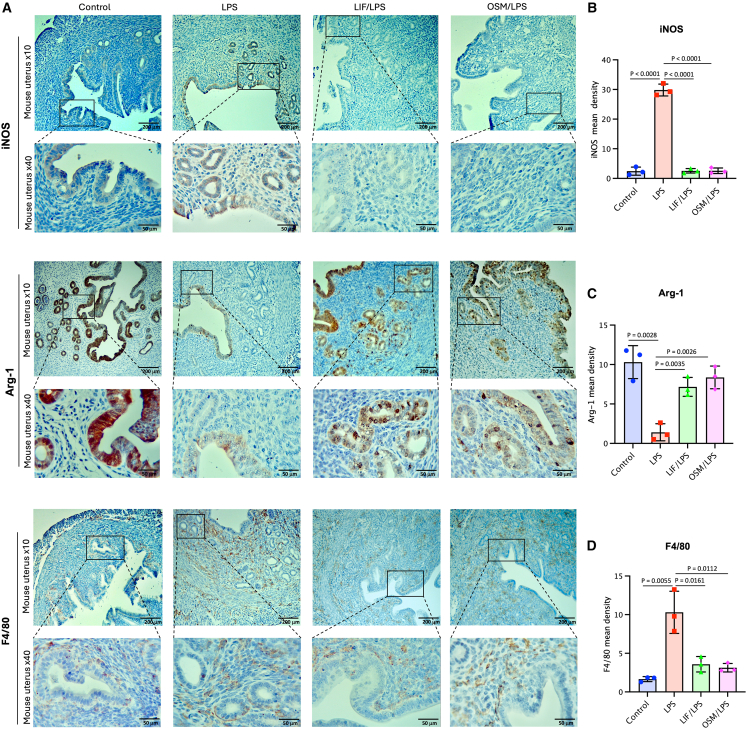
LIF and OSM modulate endometrial inflammatory response, leading to a reduction in LPS-induced endometritis (A) IHC of mice uterine tissue was used to assess immune and endocrine factor expression in the different groups. At 10× (scale bars: 200 μm) and at 40× (scale bars: 50 μm) magnification, IHC for iNOS, Arg-1, and F4/80 from the uterus were detected for the different groups. Graphical representation of iNOS (B), Arg-1 (C), and F4/80 (D) mean density per field in uterine tissue from different groups. Data are presented as mean ± SEM. Error bars represent SEM (*n* = 3 fields per group). Statistical analysis was performed using unpaired Student’s *t* test.

The levels of iNOS and Arg-1 expression in uterine tissues were evaluated by immunohistochemistry (IHC) (Figure 3A) and quantified by densitometric analyses (Figures 3B and 3C), as described in STAR Methods. We found that iNOS protein was nearly undetectable in the uterine tissues from the control group (Figures 3A and 3B); conversely, high levels of Arg-1 protein expression were seen, mainly within the uterine glands and luminal epithelium (Figures 3A and 3C). However, this protein expression pattern was completely reversed following LPS administration. The control group exhibited an iNOS mean density of 2.4 ± 1.4, whereas the LPS-treated group showed a significantly higher mean density of 29.8 ± 2.0, indicating a roughly 12-fold increase (Figure 3B). Moreover, in the LPS-treated group, Arg-1 protein expression was virtually undetectable within the uterine glands and very low in the luminal epithelium (Figure 3A). This was demonstrated by an Arg-1 mean density of 10.3 ± 2.1 in the control group, compared with a significantly lower mean density of 1.4 ± 1.1 in the LPS group, indicating a substantial 7-fold decrease in Arg-1 expression with LPS treatment (Figure 3C). Notably, the protein pattern changes induced by LPS were completely reversed by LIF and OSM injections, resulting in the total suppression of iNOS expression and a near-complete restoration of Arg-1 expression (Figures 3A and 3C).

Increased number of uterine Mϕs is another key immune response induced by LPS injection, which reflects levels of inflammation and endometritis features.^5^ The uterine immune response triggered by LPS significantly increased Mϕs, as evidenced by the elevated detection of the F4/80+ Mϕ marker (Figures 3A and 3D). The control group had an F4/80+ mean density of 1.6 ± 0.3, while the LPS-treated group displayed a substantially higher mean density of 10.3 ± 2.7, indicating an approximate 6-fold increase (Figure 3D). Additionally, in the LPS-treated group, Mϕs were localized predominantly around the uterine gland structures within the endometrial tissue (Figure 3A), a pattern distinct from the control group. In the LIF/LPS and OSM/LPS groups, Mϕ presence was reduced significantly compared with the LPS group, with F4/80 mean densities of 3.6 ± 1.0 and 3.1 ± 0.6, respectively, also reflecting a non-significant 3-fold increase compared with the control group (Figure 3D).

In summary, acute LPS exposure drives uterine iNOS expression and macrophage accumulation, contributing to tissue injury and inflammation. LIF and OSM abrogate these effects, enhance Arg-1 expression, and foster a reparative immune environment, highlighting their therapeutic potential in controlling uterine inflammation and preserving tissue integrity.

### LIF and OSM sustain the population of peritoneal Mϕs during LPS-induced inflammation *in vivo* and enhance their adherence in *ex vivo* cultures

Numerous studies have identified F4/80+ Mϕs as macrophages from the peritoneal cavity. They accumulate at sites of tissue damage where they remove necrotic cells and promote revascularization to facilitate the resolution of tissue damage.^33^ Also, while macrophages in the peritoneal cavity provide immune surveillance of the cavity and neighboring tissues^34^ they are also implicated in many pathologies, such as endometriosis and pancreatitis.^35^

**Figure 4:**
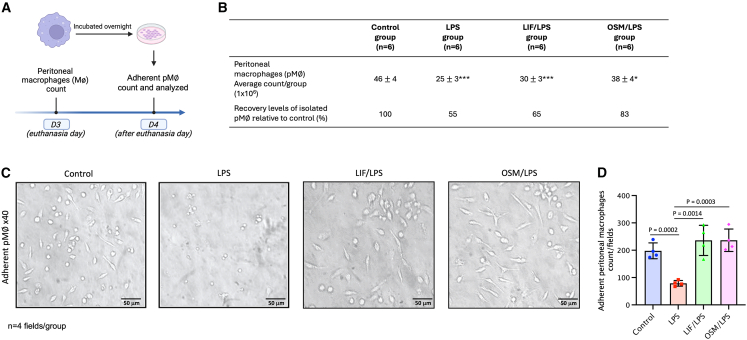
LIF or OSM treatments preserve the morphology of pMφs altered by LPS administration (A) Schematic diagram of experimental animal design and pMφ counts and morphology for the different groups. At D3 mice were euthanized and pMφs were collected, pooled per group, counted for each group, and incubated overnight. (B) Average counts of pMφs per group and percentage difference of pMφs isolated from each group compared with the control group. The control group is defined as having a baseline count of pMφs set at 100%. (C) Pictures of adherent pMφs from the different groups at D4 after an overnight incubation. (D) Graph of adherent pMφ count per field after an overnight incubation for the different groups. Data were expressed as the mean ± SEM (*n* = 4 fields per group). Data are presented as mean ± SEM. Error bars represent SEM (*n* = 4 fields per group). Statistical analysis was performed using unpaired Student’s *t* test. ∗*p* < 0.05; ∗∗∗*p* < 0.001.

We have collected, counted, and incubated pMϕs from mice of the different groups after euthanasia to determine pMϕ activity depending on the different treatments (Figure 4A). pMϕs obtained by lavage from the peritoneal cavity were considerably higher in the control group (46 ± 15 × 10^6^ from *n* = 6) than in the LPS group (25 ± 13 × 10^6^ from *n* = 6) and the LIF/LPS group (30 ± 13 × 10^6^ from *n* = 6). Compared with the control group, the LPS group exhibited a considerable reduction in pMϕs, amounting to approximately 55% of the control group’s count (Figure 4B). Similarly, the LIF/LPS group also showed a significant decrease, amounting to approximately 65% of pMϕs in the control group (Figure 4B). Notably, stimulation with OSM resulted in a higher level of pMϕs (38 ± 14 × 10^6^ from *n* = 6) than the LPS or LIF/LPS group, corresponding to approximately 83% of the control group’s count (Figure 4B).

Following the count, pMϕs were plated and incubated overnight to assess their ability to adhere within a 24-well cell culture plate depending on the different *in vivo* treatments (Figure 4C). From our microscopic analysis, we evaluated the number of adherent cells per field at 191 ± 25 cells (*n* = 4 fields) in the control group (Figures 4C and 4D). Regarding morphology, pMϕs exhibited a round or oval shape under microscopy with small extensions and no visible protrusions, suggesting they are not in an activated state nor engaged in phagocytosis or inflammatory activity.^36^ In contrast, pMϕs from the LPS group displayed an activated state, characterized by a rounded and irregular shape with protrusions and clusters indicative of apoptosis, reflecting heightened engagement in phagocytosis and inflammatory functions.^36^ This morphological shift is functionally relevant, as a rounded Mφ shape has been associated with the activation of the nuclear factor κB pathway and a pro-inflammatory phenotype.^37^ Additionally, a significant reduction was noted in the number of adherent cells per field at 79 ± 8 cells in the LPS group (*n* = 4 fields) (Figures 4C and 4D). Interestingly, pMϕs from the LIF/LPS and OSM/LPS groups exhibited distinct morphologies compared with the control group, showing a more spread appearance with elongated and fusiform shapes. Furthermore, we observed an increase in the number of adherent cells with LIF and OSM injections, with 222 ± 51 cells per field (*n* = 4 fields) for the LIF/LPS group and 240 ± 44 cells per field (*n* = 4 fields) for the OSM/LPS group. This result indicates that injections of LIF and OSM maintain the survival of Mϕs and enhance the adhesion capacity of pMϕs despite LPS treatment.

Morphological analysis of pMφs revealed distinct phenotypic alterations in response to LPS and cytokine treatments. Notably, in mice pre-treated with LIF or OSM prior to LPS administration, pMφs adopted an elongated morphology, more similar to the control group, and markedly different from the rounded appearance induced by LPS alone. The cellular shape reflects the underlying cytoskeletal organization, which governs critical macrophage functions such as migration, motility, and adhesion.^12^^,^^37^ Thus, the restoration of an elongated morphology in the LIF/LPS and OSM/LPS groups may indicate a transition toward a less inflammatory state.

In addition to morphological differences, we observed a significant reduction in the number of adherent pMφs in the LPS-treated group compared with all other groups. This decline in pMφs number could be consistent with previous reports demonstrating that LPS induces apoptosis in bone marrow-derived macrophages through two distinct mechanisms: early apoptosis mediated by autocrine TNF-α secretion and late-phase apoptosis driven by NO production.^12^ These findings complement our previous observations in uterine tissue, where LIF and OSM pretreatment reduced iNOS expression in response to LPS. These results suggest that both cytokines might protect pMφs from LPS-induced apoptosis by attenuating NO production and modulating Mφ activation status.

To further characterize the pMφ phenotypes under these inflammatory conditions, we assessed the expression of Arg-1, a hallmark of the M2 anti-inflammatory phenotype, in parallel with iNOS, the canonical marker of pro-inflammatory M1 macrophages. This allowed us to distinguish the polarization states of pMφs following exposure to LPS, and to evaluate how LIF and OSM influence their functional programming.

### LIF and OSM impact the expression of iNOS and Arg-1 in pMϕs during LPS-induced inflammation *in vivo*

**Figure 5:**
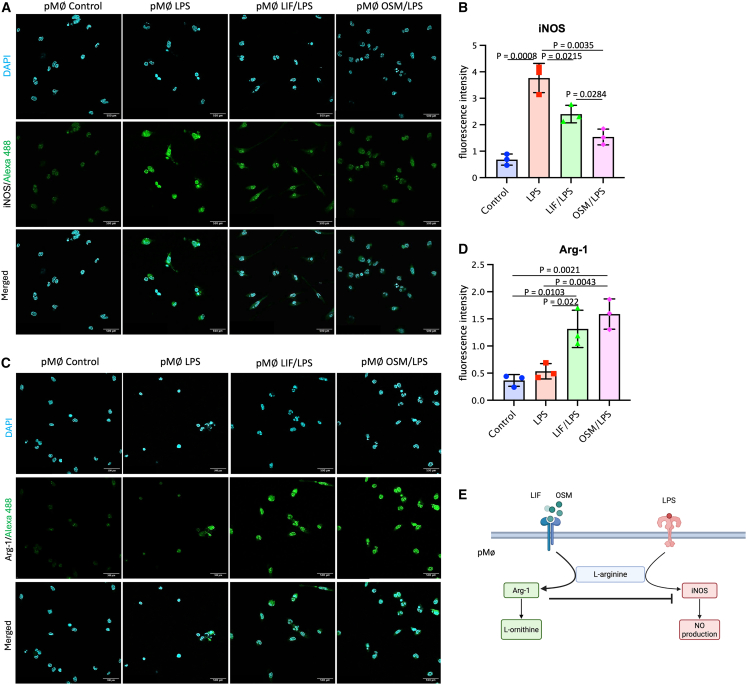
LIF and OSM modulate the LPS-induced inflammatory response by regulating arginine metabolism in pMφs Representative images of immunofluorescence detection for iNOS (A) and Arg-1 (B) proteins from mouse pMφs for the different groups. Graphical representation of iNOS (C) and Arg-1 (D) mean density per field in pMφs from the different groups. (E) Regulation of iNOS and Arg-1 expression by LPS, LIF, and OSM in macrophages. Data are presented as mean ± SEM. Error bars represent SEM (*n* = 3 fields per group). Statistical analysis was performed using unpaired Student’s *t* test. Scale bars: 500 μm.

LPS-activated Mφs express iNOS, an enzyme that metabolizes arginine into NO and citrulline to support an inflammatory response.^38^ On the other hand, to promote the resolution of inflammation, Mφs express Arg-1, which hydrolyzes arginine into ornithine and urea, thereby reducing the availability of arginine for NO synthesis. Ornithine is subsequently used as a substrate to produce polyamines and proline, both essential for cell proliferation and tissue repair (Figure 5E).^32^ The functional balance between iNOS and Arg-1 expression in macrophages is essential not only for controlling inflammation but also for defining Mϕ polarization states.^38^

Immunofluorescence analysis was conducted to assess the effects of LIF and OSM on iNOS and Arg-1 expression in LPS-activated pMφs (Figures 5A and 5B). Our results indicate that LPS significantly elevates iNOS expression (Mean Fluoresence Intensity [MFI] = 3.8 ± 0.6) compared with the control group (MFI = 0.7 ± 0.2). However, this increase was mitigated by LIF (MFI = 2.4 ± 0.3) and reduced even further in OSM-treated mice (MFI = 1.8 ± 0.6), representing a 47% decrease relative to the LPS group (Figure 5C). Unlike iNOS, Arg-1 expression remained low in the LPS group (MFI = 0.5 ± 0.1), showing levels comparable with the control group (MFI = 0.4 ± 0.1). However, Arg-1 expression significantly increased in both the LIF/LPS (MFI = 1.3 ± 0.3) and the OSM/LPS groups (MFI = 1.6 ± 0.3), indicating a shift in the arginine metabolism of pMφs (Figure 5D).

These results strongly suggest that LIF and OSM modulate arginine metabolism in pMφs during LPS-induced inflammation, potentially shaping Mφ plasticity and phenotype, to promote inflammation resolution or to enhance anti-inflammatory activity to support tissue repair in the uterine environment.

### LIF and OSM modulate the LPS-induced inflammatory response in pMϕs through iNOS, Arg-1, and COX-2 expression

In murine Mφs, cyclooxygenase-2 (COX-2), an inducible form of the COX enzymes, is known to be involved in various inflammatory responses triggered by LPS and inflammatory diseases. Moreover, the enzymatic activity of COX-2 is influenced by pro-inflammatory factors, including iNOS expression, particularly during LPS-induced inflammation in mouse Mφs.^39^^,^^40^ To further confirm the roles of LIF and OSM in pMφs during LPS-induced inflammation *in vivo* and their effect on macrophages response, we conducted PCR and Western blot analysis.

**Figure 6:**
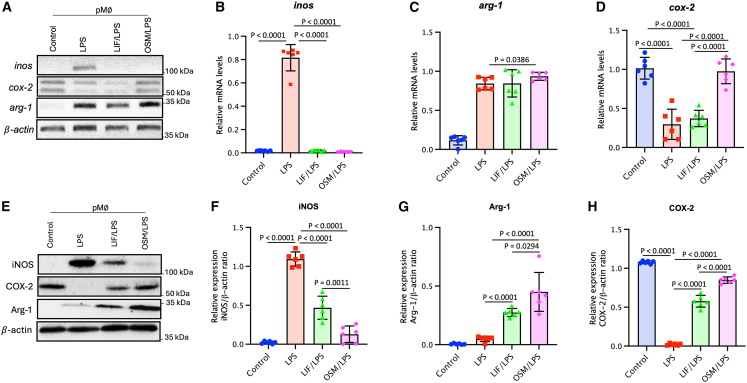
Analysis of mRNA and protein expression of enzymes associated with inflammatory activity in pMφs (A–H) pMφs from the different groups were lysed after the isolation from peritoneal cavity of mice at D3. (A) Representative images of iNOS, Arg-1, and COX-2, assessed using RT-PCR and PCR. (B–D) Graphs of relative mRNA expression of iNOS, Arg-1, and COX-2. The iNOS/*β*-actin, Arg-1/*β*-actin, and COX-2/*β*-actin ratios were determined. pMφs from the different groups were lysed with Trizol after isolation from the peritoneal cavity of mice at D3. (E) Representative images of iNOS, Arg-1, and COX-2 detection by Western blot. The iNOS/*β*-actin, Arg-1/*β*-actin, and COX-2/*β*-actin ratios were determined. Data are presented as mean ± SEM. Error bars represent SEM (*n* = 6 per group). Statistical analysis was performed using unpaired Student’s *t* test.

PCR analysis validated a substantial reduction of 4.1 and 8.3-fold of *inos* mRNA expression in pMφs from the LIF/LPS and OSM/LPS groups, respectively, compared with the LPS group (Figures 6A and 6B). The *arg-1* mRNA expression increased approximately 7.5-fold in the LPS, LIF/LPS, and OSM/LPS groups compared with the control group (+7.2× for both the LPS and the LIF/LPS groups, and +8.0× for the OSM/LPS group). However, compared with LPS and LIF/LPS injections, OSM injection induced a significantly greater increase in Arg-1 expression (Figures 6A and 6C). Surprisingly, the fold induction of *cox-2* varied among LIF and OSM stimulations. C*ox-2* expression in the LIF/LPS group, showing no significant difference with the LPS group. Conversely, the OSM/LPS group exhibited a significant 2.9-fold increase in *cox-2* mRNA induction compared with both the LPS and LIF/LPS groups, reaching levels comparable with the control group (Figures 6A and 6D).

Western blot analysis confirmed the PCR results. As shown in Figure 6E, the relative iNOS expression in pMφs was increased significantly in the LPS group (iNOS/β-actin ratio = 1.09 ± 0.09) compared with the control group (0.02 ± 0.02), representing a 49-fold induction. However, the presence of LIF or OSM significantly reduced this enhancement of iNOS expression (Figures 6E and 6F) the iNOS/β-actin ratio being 0.47 ± 0.15 (21-fold induction) in the LIF/LPS group, and 0.13 ± 0.01 (6-fold induction) in the OSM/LPS group. Compared with the control group, the relative Arg-1 expression was slightly but significantly increased in the LPS group, and even more so with either LIF or OSM injections (Figures 6E and 6G). In contrast to iNOS and Arg-1, the relative high COX-2 expression in the control group (1.08 ± 0.02) was strongly reduced in the presence of LPS (Figures 6E and 6H) but substantially restored in the LIF/LPS group (0.58 ± 0.08) and the OSM/LPS group (0.85 ± 0.04). These data confirm that LIF and OSM influence arginine and NO metabolism by decreasing iNOS expression and promoting Arg-1 and COX-2 expression in pMφs during the LPS inflammatory response.

Remarkably, treatment with either LIF or OSM restored COX-2 expression to levels comparable with those of the control group. Given the unexpected nature of this result, we further investigated the underlying molecular and cellular mechanisms by which LIF and OSM exert this regulatory effect, aiming to determine whether anti-inflammatory pathways contribute to the observed modulation.

### LIF and OSM induce anti-inflammatory signaling pathways in LPS-activated peritoneal Mϕs *in vivo*

Mφs exposed to LPS undergo classical activation, resulting in M1 polarization, which is marked by the production of pro-inflammatory cytokines and factors, including increased NO production as observed by elevated iNOS levels, a key player in pro-inflammatory signaling pathways.^41^ Conversely, the production of L-ornithine and polyamines via the arginase pathway leads to M2 Mφ polarization, characterized by the secretion of anti-inflammatory cytokines and intracellular factors.^41^ We assessed the induction of anti-inflammatory signaling pathways and immunomodulatory factors in pMφ using Western blot. Mφ polarization is modulated by many transcription factors, with STAT3^42^ and SMAD2^43^ playing a dominant role in anti-inflammatory effects. STAT3 is a primary transcription factor for M2 polarization, and JAK2/STAT3 activation results in the production of anti-inflammatory factors such as IL-10 and Arg-1.^9^ Therefore, phosphorylated STAT3 (p-STAT3) and SMAD2 (*p*-SMAD2) protein levels were assessed.

**Figure 7:**
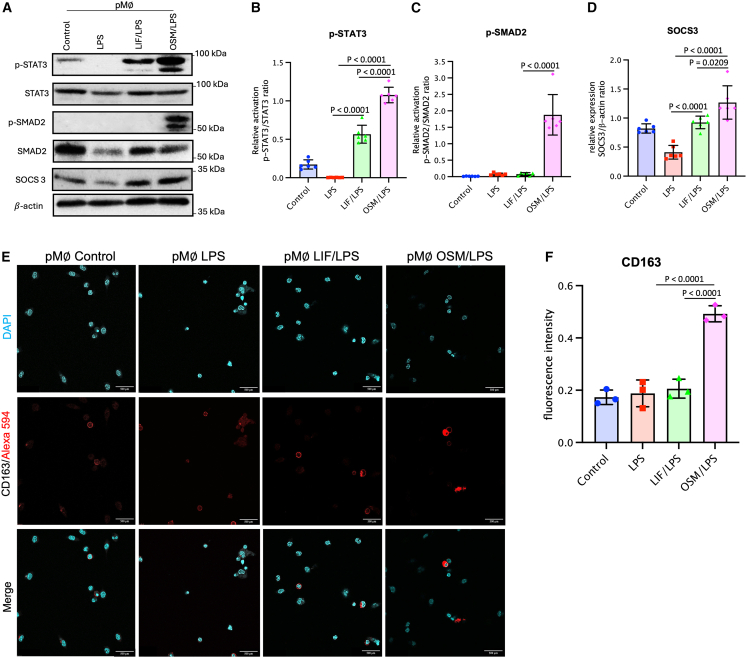
Analysis of anti-inflammatory signaling pathways regulated in pMφs (A–D) pMφs from the different groups were lysed after isolation from the peritoneal cavity of mice at D3. (A) Representative images of phosphorylated (p) and total (t) STAT3 and SMAD2, and SOCS3 as assessed by Western blotting. Graphical analysis of relative expression of p-STAT3 (B), p-SMAD2 (C), and SOCS3 (D). The p-STAT3/STAT3, p-SMAD2/SMAD2, and SOCS3/*β*-actin ratios were determined. (E and F) Analysis of surface marker expression on pMφs isolated from the peritoneal cavity of mice at D3. (E) Immunofluorescence detection of CD163 in pMφs from the different groups. (F) Graph of CD163 mean density per field in pMφs from the different groups. Data are presented as mean ± SEM. Error bars represent SEM (*n* = 6 per group for Western blot analyses; *n* = 3 fields per group for immunofluorescence). Statistical analysis was performed using unpaired Student’s *t* test. Scale bars: 500 μm.

Control, LIF/LPS, and OSM/LPS pMφs showed detectable levels of p-STAT3 (Figure 7A). pMφs from the OSM/LPS group displayed a significantly higher upregulation of p-STAT3, compared with the LPS and control groups, with the OSM group showing a 351-fold increase over the LPS group (Figures 7A and 7B). Moreover, among the different groups, a relatively higher activation of *p*-SMAD2 was induced exclusively with OSM injection (Figures 7A and 7C). Noticeable, LPS strongly decreased t-SMAD expression, but its relative expression was substantially restored in the presence of LIF and OSM (Figures 7A and 7C). pMφs isolated from LIF/LPS and OSM/LPS-treated mice exhibited significantly elevated levels of Suppressor of Cytokine Signaling 3 (SOCS3), a critical negative feedback regulator of pro-inflammatory cytokine signaling. SOCS3 expression increased approximately 1.5-fold compared with controls and nearly 3-fold compared with the LPS-only group (Figures 7A and 7D). This demonstrates that LIF and OSM enhance intrinsic mechanisms that limit excessive inflammatory responses.

In LPS-induced acute inflammation, TLR-4 receptor activation leads to iNOS expression, and STAT3 phosphorylation can inhibit this pro-inflammatory factor.^44^ Our findings suggest that LIF and OSM can shift Mφs from a pro-inflammatory toward an anti-inflammatory state via STAT3.^16^ Our data further indicate that OSM not only activates STAT3 but also engages additional anti-inflammatory signaling pathways, notably the SMAD2 cascade. SMAD2, a downstream effector of TGF-β signaling, plays a central role in immune regulation and can act with STAT3 to orchestrate anti-inflammatory effects while modulating pro-inflammatory pathways.^17^ Prior studies have also identified SMAD2 as a key mediator in suppressing iNOS expression in Mφs after LPS stimulation.^43^ The concomitant activation of STAT3, SMAD2, and SOCS3 strongly suggests that LIF and OSM reprogram Mφs toward an immunoregulatory, tissue-protective phenotype. Overall, these findings highlight the potential of LIF and OSM to dampen excessive inflammation and support tissue repair.

### OSM induces the expression of CD163, an anti-inflammatory surface marker in LPS-activated peritoneal Mϕs, compared with LIF

Since LIF and OSM activate anti-inflammatory pathways within peritoneal Mφs, we examined whether these regulatory effects can be associated with phenotypic markers associated with M2 Mφs, such as CD163. Also, M2 macrophages can be characterized by the upregulation of scavenger receptors or surface markers such as CD163.^41^ Therefore, by immunofluorescence analysis, we assessed CD163 expression in peritoneal Mφs (Figure 7E). CD163 expression levels were comparable between the control group (MFI = 0.17 ± 0.03) and the LPS group (MFI = 0.19 ± 0.05). However, a significant increase was observed exclusively in the OSM/LPS group (MFI = 0.49 ± 0.03), with the LIF/LPS group (MFI = 0.21 ± 0.04) showing levels like those in the control and LPS groups (Figure 7F). Interestingly, this suggests that OSM, in contrast to LIF, promotes an M2 Mφ phenotype characterized by CD163 expression. This cellular mechanism may involve *p*-SMAD2, which is expressed specifically with OSM injections during LPS-induced inflammation. CD163 is expressed exclusively by monocytes and Mφs,^45^ with its expression increasing in response to IL-10 and Arg-1 stimulation. Studies have indicated that CD163-related signaling modulates LPS-induced Mφs activation,^46^ and it is primarily associated with the activity of M2-polarized macrophages.^47^^,^^48^ During acute inflammation, CD163 acts as a scavenger receptor for extracellular haptoglobin-hemoglobin complexes, playing a crucial role in resolving inflammation by binding to free hemoglobin and inducing anti-inflammatory responses.^49^ Additionally, CD163 participates in the phagocytosis of apoptotic cells, helping to clear tissue debris and limiting triggers for persistent inflammation.^49^ However, the regulation of CD163 expression is still poorly understood or described. Our data suggest for the first time that the expression of CD163 could be regulated by OSM in peritoneal Mφs *in vivo* during LPS-induced acute endometritis supporting an immunosuppressive environment and contributing to inflammatory response resolution. While studies have demonstrated that OSM-mediated regulation of CD163 expression occurs in the context of chronic inflammation,^50^ our results highlight this regulatory effect in acute inflammation suggesting that the OSM/SMAD2/CD163 axis might play a role in limiting the progression of acute endometritis to chronic inflammation in uterine tissue.

## Discussion

In this study, we identify the IL-6 family cytokines LIF and OSM as potent immunoregulatory candidates capable of mitigating LPS-induced acute endometritis *in vivo*. Both cytokines markedly preserved endometrial architecture, reduced neutrophil and macrophage infiltration, and prevented the development of necrotic lesions, indicating a strong tissue-protective effect during acute uterine inflammation. These protective outcomes are mechanistically linked to the reprogramming of pMφs toward anti-inflammatory phenotypes through both shared and cytokine-specific pathways.

LPS-induced intrauterine inflammation reproduced key hallmarks of bacterial endometritis, which include edema, hemorrhage, epithelial disorganization, and dense infiltration of F4/80^+^ macrophages and inflammatory plasma cells.^5^ Consistent with previous studies, LPS robustly induced iNOS expression within the uterine tissue, reflecting the establishment of a cytotoxic microenvironment.^6^^,^^51^^,^^52^ Excessive NO production is a central driver of tissue injury and systemic complications such as vasodilation, hypoperfusion, and multi-organ dysfunction during endotoxemia.^12^ Remarkably, LIF and OSM completely abolished LPS-induced iNOS expression in both uterine tissue and pMφs. In parallel, both cytokines strongly induced Arg-1 expression, an established marker of anti-inflammatory, tissue-repairing Mφs.^38^ Through Arg-1-dependent pathways, Mφs promote collagen production, revascularization, and structural restoration of damaged endometrium,^31^^,^^38^ consistent with our histological findings.

The immunoregulatory activity of LIF and OSM is supported by the activation of STAT3 and subsequent induction of SOCS3 in pMφs, consistent with our previous observations in trophoblasts.^17^^,^^19^ STAT3-driven IL-10 production, although not directly measured here, is a well-established mechanism limiting LPS-induced TNF-α and related inflammatory mediators.^53^^,^^54^^,^^55^ SOCS3, while not directly suppressing TNF-α, plays a key role in modulating gp130-dependent cytokine signaling—particularly relevant for IL-6 family cytokines.^53^^,^^54^ Together, these pathways likely converge to stabilize a regulatory Mφ profile and attenuate downstream inflammatory cascades.

Unexpectedly, while STAT3 activation by LIF and OSM suppressed iNOS, it coincided with an increase in COX-2 expression, rather than promoting its repression. Although COX-2 is typically associated with early pro-inflammatory responses, several studies have shown that STAT3 can also induce COX-2 during the resolution phase, notably in pathways supporting vascular remodeling and angiogenesis.^56^ Based on these findings, we propose a phase-dependent regulation in which early STAT3 activity limits excessive inflammation (iNOS suppression), whereas later STAT3 (directly or via IL-10) may promote COX-2-mediated tissue repair. This hypothesis is supported by our preliminary histological observations showing more pronounced vascular structures in tissues treated with LIF or OSM.

Beyond their common effects, OSM exhibited a distinct immunoregulatory signature not observed with LIF and characterized by SMAD2 phosphorylation and substantial upregulation of CD163 in pMφs. This pathway is consistent with our previous *in vitro* observations in trophoblasts, where OSM induced both STAT3 and SMAD2 activation and increased TGF-β1 production.^17^ TGF-β1 is a canonical activator of SMAD2 and a driver of M2c macrophage polarization, an anti-inflammatory subset expressing CD163 and implicated in the resolution of inflammation.^8^^,^^57^ These data suggest that OSM may promote an OSM → STAT3/TGF-β1 → SMAD2 → CD163 regulatory axis favoring M2c-like phenotypes, particularly relevant for the late stages of inflammatory resolution. Conversely, LIF may preferentially induce an alternative anti-inflammatory subset (e.g., M2b-like), marked by CD206 rather than CD163,^8^ although additional phenotyping is required to confirm this hypothesis.

Interestingly, OSM and LIF also differed in their systemic effects, particularly at the hepatic level. While both cytokines reduced systemic inflammation and body weight loss, OSM provided a more pronounced protection against LPS-induced hepatic injury. This is consistent with the known involvement of OSM in liver regeneration and hepatoprotection, mediated by OSM-Rβ, which is highly expressed in hepatic tissues, whereas LIF-R expression is comparatively lower. Such differences may reflect divergent receptor signaling affinities as well as the added contribution of SMAD2/TGF-β1 pathways activated specifically by OSM. In support of this, Kupffer cells (F4/80^+^ liver macrophages) have been shown to secrete OSM, which is critical during the *progression phase* of liver regeneration. In a murine model of partial hepatectomy, depletion of F4/80^+^ Kupffer cells led to increased TGF-β2 levels and impaired hepatocyte proliferation, whereas reconstitution with recombinant OSM partially rescued liver regeneration.^58^ Mechanistically, Kupffer cell-derived OSM inhibits TGF-β2 signaling (via Smad2), thereby relieving a *proliferative brake* on hepatocytes and sustaining their expansion during regeneration.^58^ This evidence reinforces our observation that OSM, but not LIF, might exert superior hepatic protection not just by dampening inflammation, but also by directly promoting regenerative programs via modulation of the TGF-β axis.

Several studies have reported that LIF can transiently induce body weight loss in mice within 24–48 h post administration, likely reflecting effects on energy metabolism and lipolysis rather than overt toxicity.^24^ Indeed, LIF and OSM are established modulators of adipocyte function and lipid metabolism through the LIFR/OSMR-gp130-STAT3 signaling axis.^26^ Specifically, LIF has been shown to suppress *de novo* hepatic lipogenesis and to promote lipolysis associated with cachexia, whereas OSM exhibits a more context-dependent role, acting both as an inhibitor of adipocyte differentiation and as a regulator of white adipose tissue function in obese rodents.^25^ In line with these observations, the transient body weight reduction observed in our LIF/LPS-treated mice likely reflects a short-term metabolic adaptation rather than a sustained pro-inflammatory response. However, the underlying mechanisms remain to be fully elucidated. Future studies examining the regulation of key metabolic mediators, such as PPARγ, a central regulator of lipid metabolism and macrophage polarization that also restrains pro-inflammatory gene expression, would provide important mechanistic insights.^59^

Beyond the context of acute infection, the relevance of LIF and OSM extends naturally to early pregnancy, where both cytokines are highly expressed at the feto-maternal interface and play essential roles in implantation, decidualization, and trophoblast differentiation. Given that subclinical or overt endometrial inflammation is a major cause of implantation failure and poor IVF outcomes, our findings raise the possibility that LIF- and OSM-driven immunoregulation could also operate in gestational settings characterized by heightened inflammatory pressure. Future studies using pregnant or hormonally synchronized models will therefore be essential to determine how these cytokines interact with the endocrine milieu, decidual macrophage subsets, and implantation-associated inflammatory mediators to sustain reproductive success.

To conclude, our study identifies LIF and OSM as complementary yet distinct regulators of uterine inflammation, effectively mitigating LPS-induced acute endometritis. Both cytokines preserve endometrial structure, limit cytotoxic inflammation, and promote macrophage-mediated tissue repair, with OSM providing superior systemic protection.

### Limitations of the study

While our findings provide important insights into the immunoregulatory roles of LIF and OSM, several limitations should be considered. First, the study was conducted in a murine model using peritoneal macrophages, which may not fully recapitulate human uterine physiology, immune responses, or the diversity of uterine-resident macrophages. Additional limitations include the use of unsynchronized, non-pregnant mice and the lack of assessment of hormonal influences or direct inflammatory markers such as CD138. Future work should focus on the temporal dynamics of LIF and OSM signaling, including during pregnancy, and on characterizing uterine and decidual macrophage diversity and immunometabolic activity to fully define their therapeutic potential. However, extrapolation of these results to human conditions should be done cautiously, as species-specific differences in cytokine signaling and macrophage polarization may influence outcomes.

**Table 2:** Sequences of PCR primers

Primers	Anti-sense (AS) sequences	Sense (S) sequences
*inos*	AS-TCCATGGTCACCTCCAACACAAGA-	S-TTCACCCAGTTGTGCATCGACCTA-
*arginase (arg-1)*	AS-AGGAGCTGTCATTAGGGACATC-	S-CTCCAAGCCAAAGTCCTTAGAG-
*cox-2*	AS-GATACACCTCCACCAATGACC-	S-CAGACAACATAAACTGCGCTT-
*β-actin*	AS-TTTGGGGGATGTTTGCTCCA-	S-TGAGCTGCGTTTTACACCCT-

## Resource availability

### Lead contact

Should further information or requests for resources be required, these should be directed to the lead contact, who will ensure that they are fulfilled, Carlos Reyes-Moreno: carlos.reyes-moreno@uqtr.ca; Tel.: +1-(819)-376-5011 (ext. 3308).

### Materials availability

This study did not generate new materials or new mouse strains. All experimental mouse models used in this study are described in detail in the experimental model and study participant details section. The raw data supporting the conclusions of this article will be made available by the authors on request.

### Data and code availability

• Data: All RNA and DNA sequencing data were described in Table 2.
• Code: This paper does not report original code.
• Other: This paper does not report any additional resources.

## Acknowledgments

We thank Laurie Fortin, Nadia Desnoyers, Christel Perron, and Michel Demers for technical assistance with the animal experiments. We also thank Professor Catherine A. Thornton from Swansea University Medical School for her careful reading and constructive comments on the manuscript. This research was funded by grants from the 10.13039/501100000038Natural Sciences and Engineering Research Council of Canada (NSERC; RGPIN-2014-6516 to C.R.-M.) and the 10.13039/501100003150Fonds Québécois de la Recherche sur la Nature et les Technologies – Réseau Québécois en Reproduction (FQRNT-RQR; 2018-RS4-203214 to C.R.-M.). The RQR-CREATE scholarship program supported M.R.

## Author contributions

Conceptualization, C.R.-M.; methodology, C.R.-M. and J.G.; validation, M.R. and J.G.; formal analysis, C.R.-M. and M.R.; investigation, M.R. and J.G.; resources, J.G.; writing – original draft preparation, M.R. and C.R.-M.; writing – reviewing and editing, J.G.; supervision, C.R.-M., C.V., and C.V.T.; project administration, C.R.-M.; funding acquisition, C.R.-M. All authors have read and agreed to the published version of the manuscript.

## Declaration of interests

The authors declare no competing interests.

## Declaration of generative AI and AI-assisted technologies in the writing process

The authors acknowledge the use of Grammarly and DeepL Write for language editing support. All AI-assisted texts were reviewed and revised by the authors and external proofreaders. The authors acknowledge the use of BioRender for figure and graph creation.

The authors take full responsibility for the contents.

## STAR★Methods

### Key resources table

**Table:** 

REAGENT or RESOURCE	SOURCE	IDENTIFIER
Cell culture media, serum, and culture reagents	Wisent (St-Bruno, QC, Canada)	N/A
Cell culture plates and flasks	Corning Incorporated (Corning, NY, USA)	N/A
Bovine serum albumin (BSA)	Sigma Chemical Company (Oakville, ON, Canada)	N/A
Protease and phosphatase inhibitors cocktail EDTA-Free	Thermo Fisher Scientific (Rockford, IL, USA)	N/A
Trizol reagent	Invitrogen (Burlington, ON, Canada)	N/A
Direct-zol RNA MiniPrep Kit	Zymo Research (Burlington, ON, Canada)	N/A
Phosphate-buffered saline (PBS)	Sigma Chemical Company (Oakville, ON, Canada)	N/A
PCR primers	Invitrogen (Burlington, ON, Canada)	N/A
Taq DNA polymerase and M-MuLV reverse transcriptase	New England Biolabs (Pickering, ON, Canada)	N/A
All electrophoresis grade chemicals	Sigma Chemical Company (Oakville, ON, Canada)	N/A
Ultra Science Femto Western Substrate kit	FroggaBio (Concord, ON, Canada)	#CCH365
iNOS	Cell Signaling Technologies (Danvers, MA, USA)	#2977
Arginase-1 (Arg-1)	Cell Signaling Technologies (Danvers, MA, USA)	#93668
phospho-STAT3 (pY705)	Cell Signaling Technologies (Danvers, MA, USA)	#9145
total-STAT3	Cell Signaling Technologies (Danvers, MA, USA)	#4904
phospho-SMAD2 pS465/467	Cell Signaling Technologies (Danvers, MA, USA)	#3108
total-SMAD2	Cell Signaling Technologies (Danvers, MA, USA)	#5359
Mouse monoclonal antibodies targeting CD163	Santa Cruz Biotechnology (Santa Cruz, CA, USA)	ED2: sc-58965
Anti-mouse IgG Alexa Fluor 594	Santa Cruz Biotechnology (Santa Cruz, CA, USA)	8890S
Anti-rabbit IgG Alexa Fluor 488	Cell Signaling Technologies (Danvers, MA, USA)	4412S
Peroxidase-conjugated mouse anti-β-actin antibody	Thermo Fisher Scientific (Rockford, IL, USA)	N/A
HRP goat anti-rabbit IgG (1:5000)	Bio-Rad Laboratories (Mississauga, ON, Canada)	N/A
Recombinant LIF mouse protein	Peprotech (Montreal, QC, Canada)	N/A
Recombinant OSM mouse protein	R&D Systems (Minneapolis, MN, USA)	N/A
LPS (E. coli O55:B5)	Sigma-Aldrich (Oakville, ON, Canada)	N/A

### Experimental model and study participant details

#### Ethics statement

All animal experiments were approved by the Université du Québec à Trois-Rivières Institutional Animal Care (CBSA) and Use Committee in accordance with the guidelines of the Canadian Council on Animal Care (CCAC). The study was conducted under protocol number: 2018 - C.R.M.8; This decision is registered under protocol number CBSA-21-141-10.2.

#### Animals and experimental design

All animal experiments were conducted according to rules of the animal care and use committee of the Université du Quebec à Trois-Rivières (Trois-Rivières, Canada). Female CD-1 mice (Charles River Laboratories, Saint-Constant, Canada), weighing 27–29 g at 8–9 weeks of age, were used for the establishment of LPS-induced endometritis. The mice were housed with free access to food and water on a 12:12 h light:dark cycle with the room temperature maintained at 21°C. To induce acute inflammation, intraperitoneal (i.p.) administration of LPS at approximately 180 μg/kg body weight (5 μg of LPS per mouse) was based on an average mouse weight of 28 g. To assess the potential of LIF and OSM in protecting against LPS-induced injury, mice were distributed into four groups (*n* = 6): 1) PBS/PBS (control) group; 2) PBS/LPS group, 3) LIF/LPS group; and 4) OSM/LPS group (Figure 1A). The study was conducted under protocol number: 2018 - C.R.M.8; This decision is registered under protocol number CBSA-21-141-10.2.

### Method details

#### Peritoneal macrophage (pMϕ) isolation and cell culture

At day 3 (D3), the mice were sacrificed using pentobarbital anesthesia. Whole peritoneal macrophages were carefully isolated in cold Hank’s Balanced Salt Solution (HBSS) and kept on ice before being pooled and counted. Briefly, peritoneal cells were collected by lavage with 5 mL of HBSS. The peritoneal fluid was aspirated and transferred into a 15 mL conical polypropylene centrifuge tube kept on ice. Red blood cells (RBCs) were lysed using Ammonium Chloride Solution according to a standard protocol. The peritoneal exudate cells were then centrifuged in a refrigerated centrifuge at 400×*g* for 5 min at 4°C. The supernatant was discarded, and the cell pellet was resuspended in 2 mL of RPMI-1640 culture medium supplemented with 10% heat-inactivated fetal bovine serum (FBS), 1 mM sodium pyruvate, 10 mM HEPES, and 50 μg/mL gentamicin. Cells were then pooled by group and counted using Coomassie Blue staining to discriminate dead cells, after which each pooled sample was subdivided for the different downstream analyses and aliquoted for appropriate storage conditions. After pooling, the cells were lysed in equal volumes, and the resulting lysates were divided into several equal aliquots. This approach allowed us to perform repeated, independent analyses using separate aliquots, ensuring technical reproducibility and enabling valid statistical testing.

For morphological analysis, a subset of pMϕ was seeded in 24-well culture plates at a density of 5 × 10^5^ cells/0.5 mL in RPMI-1640 medium supplemented with 10% FBS. Cells were incubated overnight in a humidified incubator at 37°C with 5% CO_2_. After 24 h, non-adherent cells were gently removed by washing twice with warm culture medium, and adherent macrophages were examined under an optical microscope to assess cell health and morphology. All observations were performed on monolayer cultures at 20× magnification. To quantify adherent cells, five random fields were analyzed for each treatment, and representative fields are shown.

#### IHC with uterine and liver tissues

In uterine and liver tissues, quantitative protein expression of iNOS, Arg-1, F4/80 was assessed by immunohistochemistry (IHC). Briefly, 5 μm thick paraffin sections of mouse liver tissues and transverse sections of the uterus were cut. Tissue sections were dewaxed using a protocol designed to prevent the drying of blades throughout the process. Paraffin wax was removed by using xylene baths. Subsequently, rehydration was performed by immersing the sections in two baths of 100% ethanol, followed by two baths of 95% ethanol, and washing with water. For antigen unmasking, slides were placed in a citrate solution and subjected to microwave heating, until the citrate solution began to boil, the heating was maintained to keep the solution at a temperature range of 95°C–98°C for 10 min. Subsequently, the slides were allowed to cool to 30°C, then they were washed in water, incubated in 3% hydrogen peroxide for 10 min to block endogenous peroxidase activity and then washed twice with water. Slides were rinsed once in Tris-buffer saline with Tween 20 (TBST) before incubation with 100–400 μL of blocking buffer (PBS 1×, goat serum 5%, Tween 20 0.1%)/section for 60 min at room temperature. The blocking buffer was removed and 100–400 μL of primary antibody dilution was added: Arg-1 (1:100), iNOS (1:100), and F4/80 (1:300), and slides were covered by a piece of parafilm and incubated overnight at 4°C. After an incubation period of 24h, primary antibodies were removed, and slides were washed in TBST. Then, 40–120 μL of SignalStain Boost (HRP Rabbit) was added to the slides, which were subsequently incubated in a humidified chamber at room temperature for 30 min and washed again with TBST. Then, 40 μL of SignalStain DAB Substrate was prepared and applied for 10 min to the slides for signal development. Finally, slides were washed with TBST, counterstained with hematoxylin, dehydrated and mounted for analysis. IHC evaluation was performed at 10× and 40× magnification on stained tissue. Cells stained (brown) for Arg-1, iNOS, and F4/80 were quantified as the total area of positive stain in 5 random microscopic (100×) fields in each different treatment (*n* = 5), as described.^30^

Results were obtained with *n* = 6 mice per group (Control, LPS, LIF/LPS, and OSM/LPS), and each assay was performed in at least triplicate per mouse. Data are expressed as mean ± SD. Statistical comparisons between groups were performed using unpaired t-tests or Wilcoxon tests, as appropriate, with Prism software (version 9.5.0, GraphPad). A *p* value ≤0.05 was considered statistically significant.

#### Histological evaluation of uterine tissue

Uterine tissue sections were fixed, paraffin-embedded, sectioned (5 μm), and stained with hematoxylin and eosin (H&E). Histopathological scoring was performed blinded to experimental group. Five parameters were scored: epithelial integrity, glandular structure, hemorrhage, inflammatory cell (IC) infiltration, and apoptosis/necrosis. Each parameter was scored on a 0–3 scale (0 = absent/normal, 1 = mild, 2 = moderate, 3 = severe). For each animal, three representative fields per section were scored at 10× magnification, and the mean score per parameter was calculated. The total histopathology score (sum of five parameters) ranged from 0 to 15. Two independent observers scored all sections; inter-observer agreement was assessed using Cohen’s kappa. Statistical comparisons among groups were performed using unpaired *t* test or non-parametric tests where appropriate. Results are presented as mean ± SD.

#### Histological evaluation of liver tissue

Liver sections were fixed in 10% neutral buffered formalin, paraffin-embedded, sectioned at 5 μm, and stained with H&E. Scoring was performed blinded to experimental group. Five parameters were evaluated: mononuclear hepatocytes, inflammatory cell (IC) infiltration, sinusoid expansion, necrotic areas, and binuclear hepatocytes. Each parameter was scored on a 0–3 scale (0 = absent/normal, 1 = mild, 2 = moderate, 3 = severe). For each animal, three representative fields per section were scored at 10× magnification, and the mean score per parameter was calculated. The total histopathology score (sum of five parameters) ranged from 0 to 15. Two independent observers scored all sections; inter-observer agreement was assessed using Cohen’s kappa. Statistical comparisons among groups were performed using unpaired *t* test or non-parametric tests where appropriate. Results are reported as mean ± SD.

#### Protein immunodetection

After isolating pMϕ from mice treated as described above, protein expression was analyzed by Western blot. A portion of the pooled peritoneal macrophages was lysed by adding 200 μL of pre-boiled (95°C) SDS solubilization buffer containing 1.25 mM Tris-Base pH 6.8, 4% SDS, 10% β-mercaptoethanol, 18% glycerol, 0.03% bromophenol blue, and 2% protease and phosphatase inhibitors. Protein samples were resolved using SDS-PAGE, under reducing conditions and then transferred to the PVDF membrane. Blots were probed with rabbit polyclonal primary antibodies against total (t) or phosphorylated (p) forms of STAT3 and SMAD2 proteins and against the enzymatic proteins iNOS, COX-2 and SOCS3 at 1:1000 dilution at 4°C overnight. Membranes were then incubated with HRP-conjugated goat anti-rabbit IgG antibody (1:3000 dilution) for 1 h at room temperature. β-actin (1:40,000 dilution) was used as a loading control. Protein bands were visualized using an image analysis system (Alpha Innotech FluorChem FC2 Imaging System).

#### RNA detection and mRNA quantification by PCR

After isolating total cellular RNA from pMϕ, mRNA quantification was performed as previously described.^19^ Briefly, 3 × 10^6^ of pooled peritoneal macrophages were lysed using TRIzol (In Vitrogen, Montreal, QC, Canada), and cellular RNA was isolated using a Direct-zol RNA MiniPrep Kit (Zymo Research, Burlington, ON, Canada). The mRNA expression profile was evaluated using standard reverse transcription polymerase chain reaction (RT-PCR). The primers used to assess target gene expression are listed in Table 1 β-actin mRNA was used as an internal control. PCR conditions were selected to ensure amplification occurred within the exponential phase, thereby avoiding amplification near the plateau or saturation phase.

#### Immunofluorescence staining and detection

Briefly, cells were plated at a cell density of 2.0 × 10^5^ cell/2 mL/well on coverslips in 6-well plates and incubated overnight at 37°C in RPMI-1640 supplemented with 10% FBS. Cells were then fixed with 1 mL of formalin/well for 15 min at room temperature. Fixed cells were washed twice with PBS, and permeabilized with 1 mL/well of ice-cold methanol for 15 min at −20°C. Permeabilized cells were washed twice with PBS and incubated for 1 h at room temperature with 50 μL/coverslip of blocking buffer (PBS 1×, goat serum 5%, Triton X-100 0.1%), covered by a piece of parafilm to avoid evaporation. Thereafter, cells were washed with 1 mL of PBS and incubated with 50 μL/coverslip of IgG anti-goat antibodies (1:1000) for 1 h at room temperature to mask Fc binding sites on macrophages. Then, cells were washed with PBS before being incubated overnight at 4°C with primary antibodies: Arg-1 (1:50), iNOS (1:50), and CD163 (1:50). After, cells were rinsed twice with PBS, and incubated for 30 min at room temperature with a fluorescent secondary antibody: goat anti-rabbit Alexa 488 (1:1000) or goat anti-mouse Alexa 594. Finally, nuclei were stained using Prolong Gold + DAPI. Cells were viewed using an Axio Observer inverted microscope (Carl Zeiss MicroImaging, Göttingen, Germany). All observations were performed at 20× magnification on cell monolayers. Cells stained for Arg-1, iNOS, and CD163 were quantified as the intensity of positive stain in 5 random microscopic (100×) fields in each different treatment (*n* = 5).

### Quantification and statistical analysis

Each result was obtained with *n* = 6 mice per group (Control, LPS, LIF/LPS, and OSM/LPS), and each assay was performed in at least triplicate per mouse. Data are presented as mean ± SEM. Statistical comparisons between groups were performed using unpaired t-tests or Wilcoxon tests, as appropriate, with Prism software (version 9.5.0, GraphPad). A *p value* ≤ 0.05 was considered statistically significant. Statistical analysis for Figure 4 was performed using an unpaired Student’s *t* test, with significance indicated as ∗*p* < 0.05; ∗∗∗*p* < 0.001; ns, not significant.

## Supplemental information

Data S1. Mice_BW_analysis_related to Figures 1B and 1C

Data S2. ICs count_livers_mice LPS-LIF-OSM_related to Figure 2C

Data S3. ICs_count_uterus_mice LPS-LIF-OSM_related to Figure 2A

Data S4. Macrophages count_datasheets_related to Figure 4C

Data S5. Uterus_datasheets_related to Figure 2

Data S6. Liver_datasheets_related to Figure 2

Data S7. IHC_datasheets_related to Figures 3A–3E

Data S8. IF_datasheets_related to Figure 7F

Data S9. PCR_images Original PCR gel images supporting mRNA expression analyses presented in Figure 6A.

Data S10. WB_uncropped_ replicates Original uncropped Western blot images supporting protein expression analyses are presented in Figures 6E and 7A.
